# Mental Health Literacy Programs for Parents of Adolescents: A Systematic Review

**DOI:** 10.3389/fpsyt.2022.816508

**Published:** 2022-05-02

**Authors:** Sakurako Kusaka, Satoshi Yamaguchi, Jerome Clifford Foo, Fumiharu Togo, Tsukasa Sasaki

**Affiliations:** ^1^Department of Physical and Health Education, Graduate School of Education, The University of Tokyo, Tokyo, Japan; ^2^Research Fellow of Japan Society for the Promotion of Science, Tokyo, Japan; ^3^Department of Genetic Epidemiology in Psychiatry, Central Institute of Mental Health, Medical Faculty Mannheim, University of Heidelberg, Mannheim, Germany

**Keywords:** adolescents, children, mental health literacy, parents, program evaluation

## Abstract

**Introduction:**

Many mental illnesses begin during adolescence. Parents of adolescents need to have sufficient mental health literacy (MHL) to recognize mental health problems in their children and to assist them with help-seeking. Although several educational programs have been developed to enhance parental MHL, their effectiveness has not been established. This study provides a systematic review for the effectiveness of MHL programs in parents of adolescents.

**Methods:**

PubMed, PsycINFO, CINAHL, ERIC and Web of Science were searched from the earliest date possible until February 2022; references of studies which met eligibility criteria were also screened. Studies that assessed quantitative change in at least one of the following components of parental MHL were included: knowledge of mental health/illnesses; stigma toward people with mental health problems; confidence in helping children with mental health problems, and intention, knowledge or behavior of helping children with mental health problems. Risk of bias (ROB) for each outcome within the included studies was rated using the revised Cochrane risk-of-bias tool for randomized trials for randomized controlled trials (RCTs), and the Risk of Bias Assessment Tool for Nonrandomized Studies for nonrandomized studies.

**Results:**

Nine studies (four RCTs, three controlled before-and-after studies, and two case series), reported in 10 articles, were included. Mental health knowledge and/or confidence was significantly improved in several studies, while no studies observed significant improvement in stigma and/or intention/behavior of helping children. ROB was high in five out of nine studies (10 out of 18 outcomes) and unclear in the others.

**Conclusions:**

A limited number of studies have evaluated effects of MHL program in parents and inconsistent quality contributes to difficulty in establishing their overall effectiveness. More studies with appropriate methods of recruitment, measurement and analysis, and transparent reporting are needed.

**Systematic Review Registration:**

https://www.crd.york.ac.uk/prospero/display_record.php?ID=CRD42020193072, Identifier: CRD42020193072.

## Introduction

The first onset of mental illness usually occurs during adolescence (1). However, adolescents may have difficulty in recognizing their own mental health problems (2), and even if they are aware of these problems, they may be reluctant to seek professional help (2). The majority of adolescents might think that family can help them with mental health problems (3) and ask for help from their family members when needed (4). Therefore, parents need to be able to assist their children in recognizing mental health problems and seeking appropriate help.

To assist their children with mental health problems, parents need good mental health literacy (MHL), which is knowledge and beliefs about mental disorders that aid in their recognition or prevention (5). MHL has several components such as: the ability to recognize mental disorders, knowledge of treatments available, attitudes that promote recognition of mental health problems and appropriate help-seeking, and skills to support others with mental health problems (5, 6). The ability to recognize mental disorders may be necessary to know when it is time to seek help. When it comes to seeking appropriate help, knowledge about professional help and treatments available will be useful (6). Since those who are experiencing a mental disorder may not be aware of their situation, people around them such as family members may need skills to listen to and support them to facilitate recognition and help-seeking (6).

A number of studies have assessed MHL in parents, finding that parental MHL is generally limited (7). Parents may not have adequate knowledge about the causes, symptoms (8, 9), and treatments (3) of mental health problems, resulting in difficulties recognizing mental health problems in their own children (10, 11). People who have strong stigmatizing attitudes about mental illness (9, 12) and low confidence in helping others with mental health problems (13) can be less likely to provide appropriate support (14, 15). In addition, by delaying recognition of mental health problems, inadequate MHL might be a barrier preventing parents from seeking help for their children (16, 17).

Parents may also need to have better knowledge of the MHL needs of their children. Recent work highlights these needs, which includes components such as knowledge of mental health professionals and of how to seek mental health information (18, 19). Also, reduction of stigma, and the ability to recognize common mental illness or changes in their own mental health are suggested to be important (18–20). Parents need to improve their own MHL while being aware that they are in a position to provide help and accurate information as trusted adults.

Thus far, several educational programs have been developed to improve MHL in parents of adolescents (21–23). Although each program has been evaluated, overall effectiveness of these programs has not been established. To date, one systematic review on parental MHL has been published mainly reviewing cross-sectional and qualitative studies investigating parental MHL levels (7), and including only a limited number of intervention studies investigating the effects of parental MHL programs. In the present study, we conducted a comprehensive systematic review of intervention studies which measured the effects of MHL programs in parents of adolescents in the general population.

## Methods

### Protocol and Registration

The present systematic review was conducted in accordance with the Preferred Reporting Items for Systematic Review and Meta-Analyses (PRISMA) guidelines (24, 25). The review protocol was registered with the International Prospective Register of Systematic Reviews (PROSPERO) (CRD42020193072).

### Eligibility Criteria

#### Inclusion Criteria

Our review included studies which examined the effectiveness of MHL educational programs in parents of adolescents (preteens and teenagers) in the general population, regardless of study design. Studies were included when they met both of the following two criteria.

1. They implemented programs aimed at improving literacy about mental health problems which start to increase in prevalence during adolescence. Specifically, we included programs addressing mood and anxiety disorders or related problems, which are the two most prevalent types of mental illness (1), and schizophrenia or related problems, where severe aftereffects occur and longer untreated durations are found to predict poor outcomes (26);
2. Quantitative change was assessed in at least one of the following four components of MHL in parents: (a) knowledge of mental health/illnesses and their treatments, (b) stigmatizing attitudes toward people with mental health problems, (c) confidence in helping children with mental health problems, and (d) intention, knowledge or behavior of helping children with mental health problems.

Studies with any comparison condition (e.g., no intervention, waitlist and other health education interventions) and studies using any measurement methods were included. Doctoral dissertations as well as studies from peer-reviewed journals were included, if they were written in English.

#### Exclusion Criteria

Studies were excluded when they met any of the following: (1) Baseline measurements were not conducted; (2) Studies that tested programs which exclusively targeted parents of adolescents suffering from physical illnesses or mental illnesses. Also, studies which tested programs exclusively for suicide prevention were excluded, because they have already been systematically reviewed (27).

### Study Selection

PubMed, PsycINFO, CINAHL, ERIC and Web of Science were searched from the earliest date possible until February 2022. With the exception of PubMed and Web of Science, these databases were searched via EBSCO. Search terms included were: “parent”, “mental health”, “literacy”, “young people”, “program evaluation” and other related terms as below. In addition, the reference lists of included studies were scrutinized to identify any relevant publications according to eligibility criteria.

Search terms: (parent^*^ OR family) AND (“mental disorder” OR “mental health” OR “mental illness” OR depression OR “mood disorder” OR “affective disorder” OR “anxiety disorder” OR psychosis OR schizophrenia OR “substance abuse”) AND (literacy OR belief^*^ OR attitude^*^ OR perception^*^ OR stigma OR competen^*^ OR abilit^*^ OR capabilit^*^ OR confiden^*^ OR know^*^ OR identif^*^ OR aware^*^ OR recogni^*^) AND (intervention^*^ OR “health education” OR “training” OR “teaching”) AND (adolescen^*^ OR child^*^ OR “young adult” OR “young people” OR teen^*^ OR “young person”) AND (“program evaluation” OR “program development” OR assessment OR test OR trial OR effective OR effic^*^).

Two reviewers (S.K. and S.Y.) independently screened the titles and abstracts, and excluded studies not relevant to the topic of interest. They independently reviewed the full-texts of the articles for final selection of included studies. A third reviewer (T.S.) was invited to resolve disagreements between the two reviewers.

### Data Extraction

The first author (S.K.) extracted the following data from included studies: study design, country, comparison condition, sample size, timing of data acquisition, targeted age of children, participant baseline characteristics, details of intervention (i.e., delivery mode, contents of intervention, and schedule), outcome measures, outcome data and participation rates. We attempted to contact authors of included studies when they did not report all of this information. The second author (S.Y.) confirmed the extracted data. A third reviewer (T.S.) was invited to resolve any disagreements between S.K. and S.Y.

### Risk of Bias in Individual Studies

Risk of bias (ROB) was rated for each outcome in each included study. The revised Cochrane risk-of-bias tool for randomized trials (RoB2) (28) was used to assess ROB for RCTs. The following five domains were rated as “low ROB”, “some concerns”, or “high ROB”, for each outcome of each RCT: (1) randomization process; (2) deviations from intended interventions; (3) missing outcome data; (4) measurement of the outcome; and (5) selection of the reported result. An overall ROB was rated for each outcome across the five domains according to RoB2 (28) as follows: the overall ROB was rated as “low” when ROB in all domains were rated as “low”; the overall ROB was rated as “some concerns” when ROB in at least one domains was judged to have “some concerns”, but not as “high ROB” in any domain; the overall ROB was rated as “high”, when ROB in one or more domains were rated as “high”, or when ROB in multiple domains were judged to have “some concerns” in a way that substantially lowers confidence in results.

The Risk of Bias Assessment Tool for Nonrandomized Studies (29) was used for nonrandomized studies. The following six domains were rated as “low”, “high”, or “unclear” for each outcome of each nonrandomized study: (1) selection of participants; (2) confounding variables; (3) measurement of exposure; (4) blinding of outcome assessments; (5) incomplete outcome data; and (6) selective outcome reporting. The overall ROB was rated for each outcome based on ROB in the 6 domains, according to the Risk of Bias Assessment Tool for Nonrandomized Studies (29).

Two reviewers (S.K. and S.Y.) independently rated these domains. When the judgment was different between the two reviewers, they discussed with the third reviewer (T.S.) to reach a consensus.

### Calculation of Effect Size

Within-group effect sizes (standardized mean difference [SMD] for continuous variables, odds ratios for dichotomous variables) were calculated for each of the intervention groups and control groups as follows (30):


$$\begin{array}{l} \frac{{Mean}_{post}-{Mean}_{pre}}{{SD}_{pre}} \end{array}$$

In this calculation, the denominator is the standard deviation at pre-test, and numerator is the mean score at post-test minus the mean score at pre-test. When follow-up tests were conducted in the included studies, SMD was calculated by replacing mean score at post-test by mean score at follow-up test. Effect sizes are considered to be small, medium, and large, when SMD is between 0.2 and 0.5, between 0.5 and 0.8, and over 0.8, respectively (31).

### Data Synthesis

We did not conduct a meta-analysis of results, because methodological and clinical heterogeneity was high across the included studies and no studies had low ROB (see results section). Publication bias was also not assessed. We instead present a narrative synthesis for each of the following four outcomes: (a) knowledge of mental health/illnesses and their treatments; (b) stigmatizing attitudes toward people with mental health problems; (c) confidence in helping children with mental health problems; and (d) intention, knowledge and behavior of helping children with mental health problems.

## Results

### Study Selection

**Figure 1:**
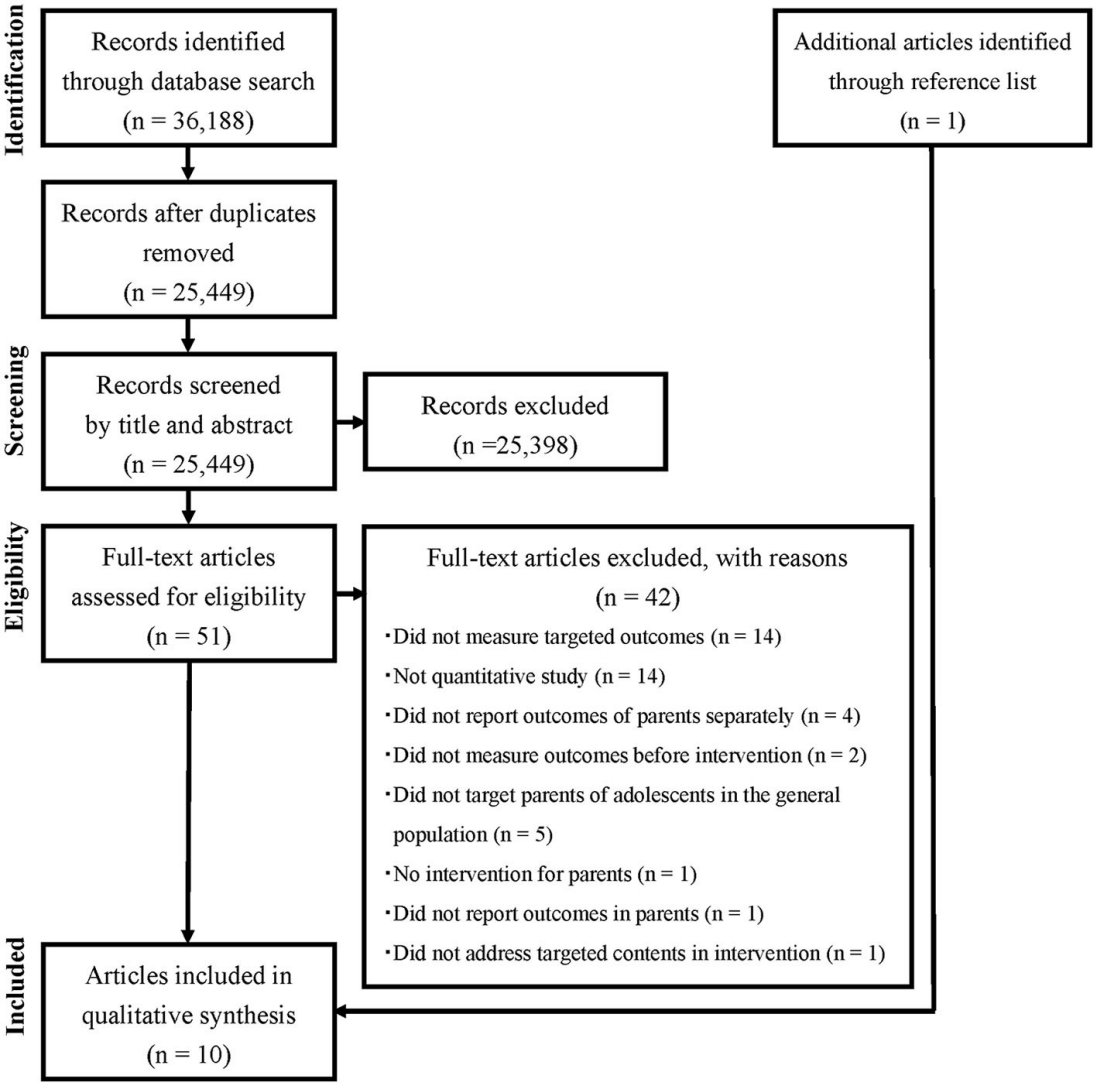
Flow chart of article selection procedure.

Figure 1 describes the flow of article selection in the present systematic review (24, 25). Electronic database searches yielded a total of 36,188 articles. After removing 10,739 duplicates, 25,449 articles remained. Of these, 25,398 articles were excluded after screening titles and abstracts. After assessing the full-texts of the remaining 51 articles, nine articles met the inclusion criteria. None of these met the exclusion criteria. The reference lists of these nine articles were screened, and one additional article which met the eligibility criteria was found. In total, 10 articles met the eligibility criteria.

### Study Characteristics

Table 1 presents characteristics of the included studies. We attempted to contact seven authors for missing information; four of them responded. The 10 articles reported the results of nine studies; results of “knowledge” (23) and “stigma” (38) from a single study were reported in different articles; for another, results of the 1- and 2-year follow-up (33), and those of the 3-year follow-up (34) were reported in different articles. The nine studies investigated effects of eight different educational programs; two studies of the same program in different samples were reported in one article (23). Four studies were randomized controlled trials (RCTs) (21, 32–35), three were controlled before-and-after studies (CBAs) (22, 36, 37) and two were case series (23, 38).

**Table 1:** Characteristics of included studies.

**Author (year)** **[country]**	**Comparison** **condition**	**Sample** **size**	**Parental age** **(Mean [SD])**	**Female** **proportion (%)**	**Targeted age** **of children**	**Details of intervention**			**Reported outcomes^a^**			
						**Delivery mode** **(face-to- face or online)**	**Teaching modalities**	**Schedule** **(intervention period and/or program length)**	**Knowledge**	**Stigma**	**Confidence**	**Help**
**Randomized controlled trial (RCT)**												
Chu et al. (2019) (21) [New Zealand]	No intervention^b^	pre: 221 post: 211 2 mo: 201	NR	96.8	10–15	Online	Text message	4 weeks^c^	+	–	–	–
Deitz et al. (2009) (32) [NR]	Waitlist	pre: 99 post: 96	Total: 42 [NR]	45.5	5–21	Online	Multimedia rich, fully narrated, and interactive modules	2 weeks^d^	+	–	+	–
Morgan et al. (2019) (33, 34)^e^ [Australia]	Red Cross Provide First Aid^f^	pre: 322 1 y: 208 2 y: 178 3 y: 149	Int: 45.2 [5.54]; Con: 45.1 [5.69]	88.2	12–15	Face-to-face	^g^ ^,^ ^h^	2 days (3.5 hours × 4 sessions in total)	+	+	+	+
Seibert (2001) (35) [USA]	No intervention	pre: 51 2 w: NR	NR	≥ 84.3^i^	Grade 4–6	Face-to-face	Didactic session^h^	85 min	+	–	–	–
**Controlled before-and-after study (CBA)**												
Choi et al. (2016) (36) [Korea]	Healthy diet intervention	pre: 214 post: 114 1 mo: 93	Int: 43.2 [3.1]; Con: 43.9 [4.2]	88.8	11–16	Online	Didactic session, video, assignment, feedback^j^	4 weeks (20-min media file per week^k^)	+	–	+	–
Hurley et al. (2018) (37) [Australia]	No intervention	pre: 66 1 mo: 55	Total: 44.9 [5.2]	77	Adolescents	Face-to-face	Group discussion, video^h^	1 hour	+	+	–	–
Hurley et al. (2021) (22) [Australia]	No intervention	pre: 540 1 mo: 284	Total: 47.4 [5.3]	59.4	Adolescents	Face-to-face	Group discussion, video^h^	1 hour	+	+	–	+
**Case series**												
Yoshii et al. (2011) (23, 38)^l^^,^^m^ [Japan]	–	pre: 2,690 1 w: 2,465	Total: 45.9 [4.7]	48.7	Junior and senior high school students	Online	Slides with narration	13 min	+	+	–	–
		pre: 735 1 w: 628	NR	47.1					+	–	–	–

*Con, control group; Int, intervention group; mo, month; NR, not reported; SD, standard deviation; w, week; y, year. Knowledge, knowledge of mental health/illnesses and their treatments; Stigma, stigmatizing attitude toward people with mental health problems; Confidence, confidence in helping children with mental health problems; Help, intention, knowledge and behavior of helping children with mental health problems*.

a *+: measured, –: not measured*.

b *Control group could access alternative services (no details described)*.

c *Intervention group daily received a text message (≤160 characters)*.

d *Participants were encouraged to watch the program as often as possible*.

e *The 3 y follow-up data in Morgan et al. (2020) (34)*.

f *Red Cross Provide First Aid is a 15-hour training for knowledge and skills to sustain life until professional help arrives*.

g *Youth Mental Health First Aid, a course for adults caring for adolescents (no details described)*.

h *Supplemented by reading materials*.

i *Consisted of 84.3% mothers, 7.8% fathers, and 7.8% guardians*.

j *Participants received feedback from research staff after submitting assignment and questions*.

k *One media file on mental health problems among adolescents, and others on their development and parent-child relationship*.

l *Effects on “Knowledge” and “Stigma” were reported in Yoshii et al. (2011) (23) and Ling et al. (2014) (38), respectively*.

m *Another study of the same program in different samples was also reported*.

Studies where parents of adolescents participated in interventions were included. They covered information about, or skills to recognize the signs/symptoms of mental health problems/mental illness (21–23, 32–38). Some programs also covered information on treatment (21–23, 32, 37, 38) or skills to assist adolescents to get appropriate professional help as early as possible (33, 34). Regarding the illnesses, depressive disorders and/or related problems were addressed in six programs (six studies) (21, 22, 32–34, 36, 37), and anxiety disorders and/or related problems were addressed in five programs (five studies) (22, 32–35, 37). Schizophrenia (or psychosis) was dealt with in two programs (three studies) (23, 33, 34, 38). Of these eight programs, four programs, studied in two RCTs (33–35) and two CBAs (22, 37), were delivered “face-to-face” and supplemented by take home reading materials (22, 33–35, 37); two of these four programs included workshops with a group discussion about prevention of mental health problems in adolescents (22, 37). The other four programs, studied in two RCTs (21, 32), one CBA (36) and two case series (23, 38), were delivered online. Specifically, one was a multimedia rich, narrated and interactive program (32), while the others used media files including a didactic session and video (36), slides with narration (23, 38), or text messages only via mobile phone short message service (SMS) (21). The total length of face-to-face programs ranged from a single 1-h session (22, 37) to four 3.5-h sessions run over 2 days (33, 34), while online programs ranged from a single 13-min session (23, 38) to four 20-min sessions (36). Teaching modalities and schedules of interventions were varied across the programs, except for the two programs by the same group (22, 37).

Timing of data acquisition was also varied; one out of the nine studies measured outcomes before (pre-test) and immediately after (post-test) the intervention (32). Pre- and follow-up tests were conducted in six studies (22, 23, 33–35, 37, 38), and pre-, post-, and follow-up tests were conducted in two studies (21, 36). Two studies (22, 37) by the same group used the same questionnaire. Other studies each used different questionnaires (21, 23, 32–36, 38). Some studies did not indicate whether the questionnaires had been validated in regular people (21–23, 32, 35, 37). Due to high methodological and clinical heterogeneity, we did not compare outcome data between the studies. In addition, participation rates were not reported in eight (21–23, 33–38) out of the nine included studies. Among these, six studies (21–23, 33, 34, 36, 37) did not describe the number of people who received information on recruitment of the study participants, which is needed to calculate the participation rate. Also, the presence/absence of adverse events was described only in one study, which observed no such event (33, 34).

### Risk of Bias

#### Risk of Bias of RCTs

ROBs in the four included RCTs are summarized in Table 2, for the five domains and the overall ROB, of the four outcomes. ROB for the 1st domain was rated as “some concerns” in three RCTs (21, 32, 35) and “low” in the other (33, 34). ROB for the 2nd domain was “high” in one RCT (35), because whether the analysis was by intention-to-treat or not was not clearly described. ROB for the 3rd domain was “high” in three RCTs (21, 32, 35), because the authors did not use statistical methods to avoid bias due to missing outcome data (21, 32) or whether the authors used such methods was not stated (35), and also because participants' levels of MHL might have affected whether they answered questions (21, 32, 35). ROB for the 4th domain was “high” in all 4 RCTs (21, 32–35) because the participants were aware of the group they were assigned to (intervention or control), and this awareness may have influenced responses to the self-report questions (21, 32–35). Internal consistency of the questionnaires were reported as low in some studies [Cronbach's alpha = 0.43 (35), Omega = 0.46 and 0.56 (33, 34)]. ROB for the 5th domain was “some concerns” for all outcomes (21, 32–35). Overall ROB was rated as “high” for all studies (21, 32–35), because ROB was “high” in at least one domain for each study.

**Table 2:** ROB in the five domains and overall ROB for 4 outcomes in randomized controlled trials.

**Study**	**Outcome**	**Domain**					**Overall ROB**
		**1) Randomization process**	**2) Deviations from intended interventions**	**3) Missing outcome data**	**4) Measurement of the outcome**	**5) Selection of the reported result**	
Chu et al. (2019) (21)	Knowledge						
Deitz et al. (2009) (32)	Knowledge						
	Confidence						
Morgan et al. (2019) ^a^ (33, 34)	Knowledge						
	Stigma						
	Confidence						
	Help						
Seibert (2001) (35)	Knowledge						

*“High risk of bias,” “Some concerns” and “Low risk of bias” are shaded dark gray, gray, and light gray, respectively*.

a *Results of the 3-year follow-up was reported in Morgan et al. (2020) (34)*.

#### Risk of Bias of Nonrandomized Studies

Table 3 summarizes ROBs for six domains and overall ROB in nonrandomized studies (three CBAs and two case series). ROB for the 1st domain was rated as “high” in two studies (22, 37), because participants of intervention and control groups were from different areas. ROB for the 2nd domain was “high” in three studies (23, 37, 38), because no confounding variables were controlled for in the analyses. ROB for the 3rd and 4th domains was “high” in all five studies (22, 23, 36–38), because the participants were aware of the group they were assigned to, and this awareness may have influenced responses to the self-report questionnaires. ROB for the 5th domain was “high” in two studies (36, 37) because retention rate markedly differed between intervention and control groups. ROB for the 6th domain was “high” in one study (36), because statistical results were not clearly reported. Overall ROB was rated as “high” in one study (37), and as “unclear” in the others (22, 23, 36, 38), according to the criteria of the Risk of Bias Assessment Tool for Nonrandomized Studies (29).

**Table 3:** ROB in the six domains and overall ROB for 4 outcomes in nonrandomized studies.

**Study**	**Outcome**	**Domain**						**Overall ROB**
		**1) Selection of participants**	**2) Confounding variables**	**3) Measurement of exposure**	**4) Blinding of outcome assessments**	**5) Incomplete outcome data**	**6) Selective outcome reporting**	
Choi et al. (2016) (36)	Knowledge							
	Confidence							
Hurley et al. (2018) (37)	Knowledge							
	Stigma							
Hurley et al. (2021) (22)	Knowledge							
	Stigma							
	Help							
Yoshii et al. (2011)^*a*^ (23, 38) 1st survey	Knowledge							
	Stigma							
Yoshii et al. (2011) (23) 2nd survey	Knowledge							

*“High risk of bias,” “Unclear risk of bias” and “Low risk of bias” are shaded dark gray, gray, and light gray, respectively*.

a *Effects on “stigma” were reported in Ling et al. (2014) (38)*.

### Effects on Outcomes

#### Effects on Knowledge of Mental Health/Illnesses and Their Treatments

Table 4 summarizes effects of programs on knowledge of mental health/illnesses and their treatments. Knowledge was significantly improved immediately after the intervention in one (32) out of two RCTs (21, 32). At the follow-up test, knowledge was significantly improved in two (33–35) out of three RCTs (21, 33–35), and one (37) out of three CBAs (22, 36, 37). In the CBA (36) and two case series (23) which measured effects immediately after the intervention and/or at follow-up, effects were uncertain due to unclear reporting. Recognition of the disease name was measured in vignette cases of depression, social phobia, psychosis and eating disorder in 1 RCT, without significant improvement (33, 34).

**Table 4:**
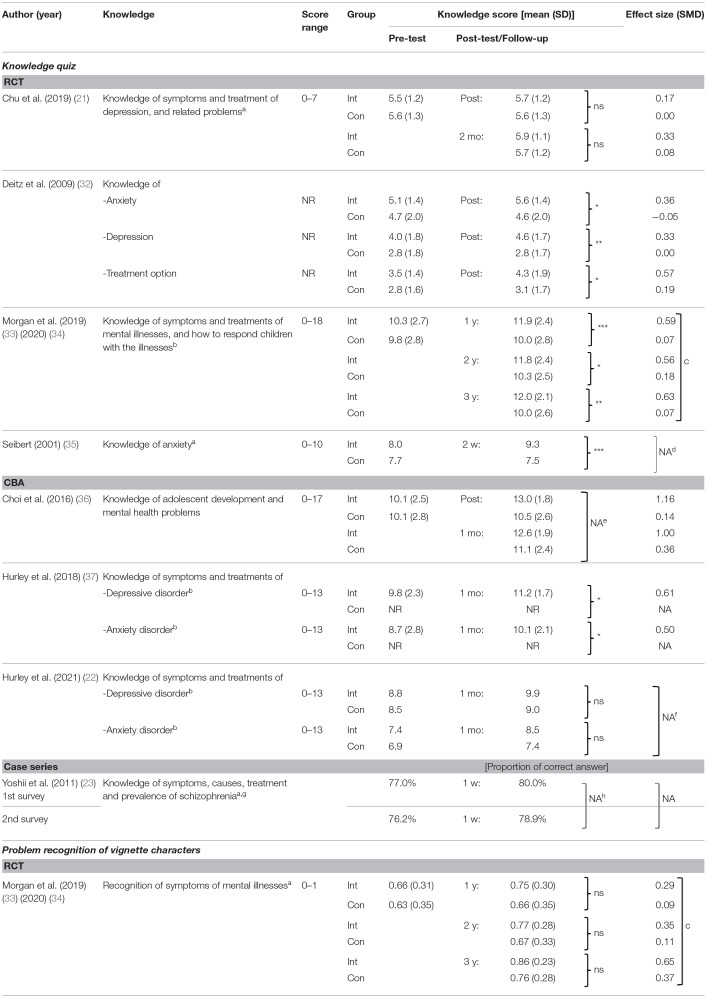
Effects on knowledge of mental health/illnesses and their treatments.

*CBA, controlled before-and-after study; Con, control group; Int, intervention group; mo, month; NA, not applicable; NR, not reported; ns, not significant; RCT, randomized controlled trial; SD, standard deviation; SMD, standardized mean differences; w, week; y, year*.

*p < 0.05, **p < 0.01, ***p < 0.001.

*^a^Questionnaire items were reported in the article*.

*^b^Questionnaire items were obtained by contacting the study authors*.

*^c^Cohen's d was 0.43, 0.26, 0.31 for knowledge quizzes, and 0.11, 0.12, and 0.07 for problem recognition of characters in vignettes (1 y, 2 y, and 3 y follow-up, respectively; change in control groups was considered)*.

*^d^We did not calculate effect size, because SD was 1 order smaller although the sample size was smaller than in other studies*.

*^e^Significance levels of statistical results were not reported*.

*^f^SD was not reported*.

*^g^The scoring method of questions to measure ability to discriminate schizophrenia symptoms was not described in the article, so results of the questions were not listed on this table*.

*^h^Unclear report of employed statistical analysis method*.

Regarding the mode of delivery, knowledge was improved in three [two RCTs (33–35), one CBA (37)] out of four face-to face programs (22, 33–35, 37) at follow-up. This improvement was also observed in one [RCT (32)] out of three online programs (21, 32, 36) immediately after the intervention.

#### Effects on Stigma Toward People With Mental Health Problems

Table 5 summarizes effects of programs on parents' stigma toward people with mental health problems. Three types of stigma were measured at follow-up in the total of four studies in five articles (22, 33, 34, 37, 38), with no significant improvement observed. The three types of stigma were as follows: (1) unwillingness to have contact with a person with mental health problems (“social distance”), (2) personal negative attitudes toward a person with mental health problems (“personal stigma”), and (3) beliefs that most people would look down on or discriminate against psychiatric patients (“perceived devaluation-discrimination”). Social distance and personal stigma were measured in 1 RCT (33, 34) and two CBAs (22, 37), which were face-to face programs, while perceived devaluation-discrimination was measured in one case series (38), an online program.

**Table 5:**
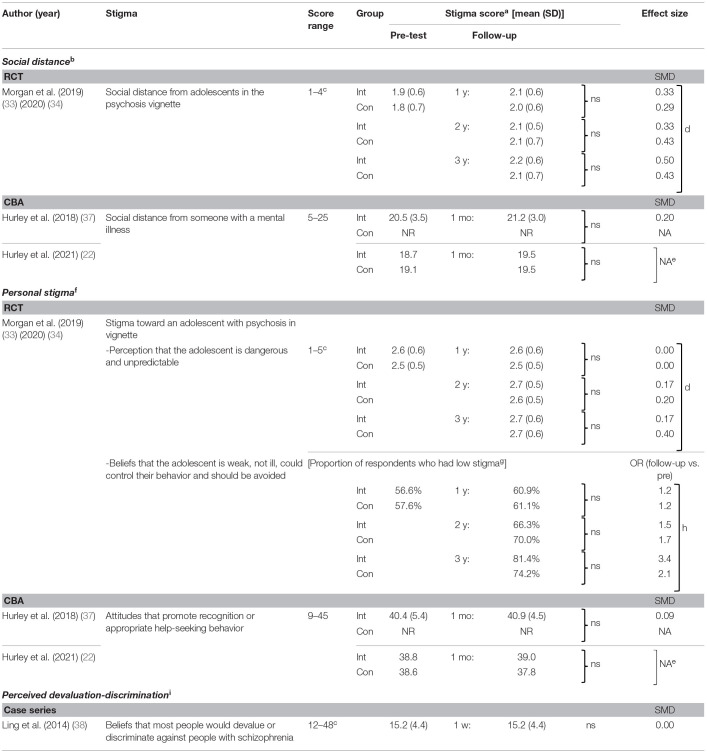
Effects on stigma toward people with mental health problems.

*CBA, controlled before-and-after study; Con, control group; Int, intervention group; mo, month; NA, not applicable; NR, not reported; ns, not significant; OR, odds ratio; RCT, randomized controlled trial; SD, standard deviation; SMD, standardized mean differences; w, week; y, year*.

*^a^Higher scores indicate lower stigma*.

*^b^Unwillingness to have contact with the person with mental health problems*.

*^c^Original scores were reversed, so that higher scores indicate lower stigma*.

*^d^Cohen's d was 0.18, −0.12, and −0.05 for “social distance,” and 0.15, 0.22, and −0.09 for “personal stigma” (1 y, 2 y, and 3 y follow-up, respectively; positive values indicating improvement by intervention; change in control groups was considered)*.

*^e^SD was not reported*.

*^f^Personal negative attitudes toward a person with mental health problems*.

*^g^1 y and 2 y follow-up data were obtained by contacting the study authors (not included in the original papers)*.

*^h^ORs were 0.94, 0.59 and 1.06 (1 y, 2 y, and 3 y follow-up, respectively; change in control groups was considered)*.

*^i^Beliefs that most people would look down on or discriminate against psychiatric patients*.

#### Effects on Confidence in Helping Children With Mental Health Problems

Table 6 summarizes effects of programs on parents' confidence in helping children with mental health problems. Confidence was measured immediately after the intervention in one RCT, with significant improvement observed (32). Confidence was measured at follow-up in another RCT, with significant improvement observed at 1-year follow-up (33), but not at 2-year (33) and 3-year follow-up (34). Confidence was also measured in one CBA (36), with uncertain results due to unclear reporting.

**Table 6:**
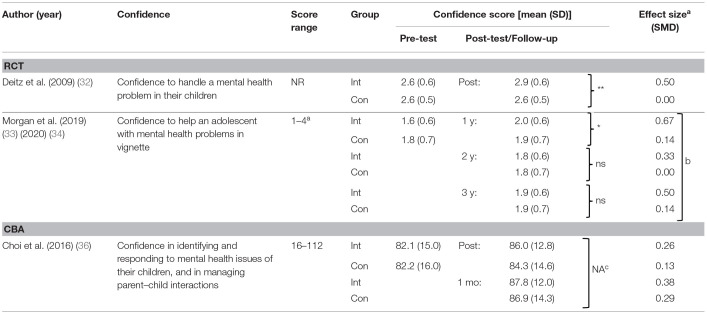
Effects on confidence in helping children with mental health problems.

*Con, control group; CBA, controlled before-and-after study; Int, intervention group; NA, not applicable; NR, not reported; mo, month; ns, not significant; RCT, randomized controlled trial; SD, standard deviation; SMD, standardized mean differences; y, year*.

*p < 0.05, **p < 0.01.

*^a^Original scores were reversed, so that higher scores indicate higher confidence*.

*^b^Cohen's d was 0.26, 0.23 and 0.01 (1 y, 2 y and 3 y follow-up, respectively; change in control groups was considered)*.

*^c^Significance levels of statistical results were not reported*.

Regarding the mode of delivery, confidence was improved in one face-to-face program at 1 year follow-up [RCT (33)]. This improvement was also observed in one [RCT (32)] out of two online programs immediately after the intervention, while the other online program [CBA (36)] had uncertain effects.

#### Effects on Intention, Knowledge and Behavior of Helping Children With Mental Health Problems

Table 7 summarizes effects of programs on parents' intention, knowledge and behavior of helping their children with mental health problems. Knowledge and behavior of helping children with the problems were measured in 1 RCT in two articles (33, 34), with significant improvements observed only for knowledge at 1 year follow-up (33). Intention to help children with the problems was measured in 1 CBA (22), without significant improvement. Both programs were delivered “face-to-face” (22, 33, 34).

**Table 7:**
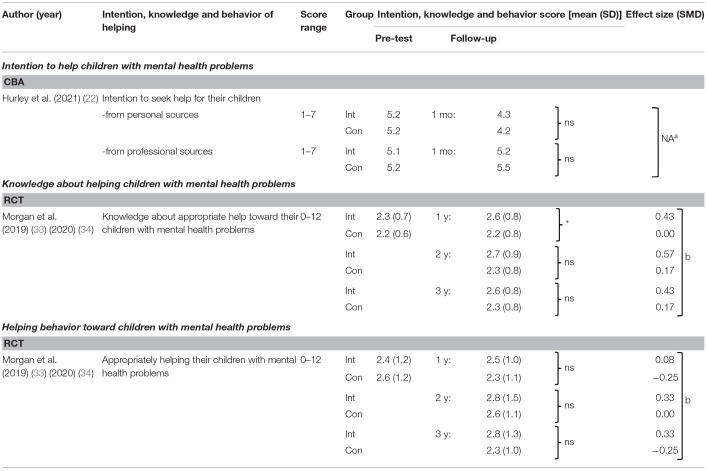
Effects on intention, knowledge and behavior of helping children with mental health problems.

*CBA, controlled before-and-after study; Con, control group; Int, intervention group; mo, month; NA, not applicable; ns, not significant; RCT, randomized controlled trial; SD, standard deviation; SMD, standardized mean differences; y, year*.

*p < 0.05.

*^a^SD was not reported*.

*^b^Cohen's d was 0.22, 0.15 and 0.09 for knowledge about helping children, and 0.17, 0.16 and 0.38 for quality of actual helping behavior (1 y, 2 y and 3 y follow-up, respectively; change in control groups was considered)*.

## Discussion

We searched studies which examined the effectiveness of MHL educational programs in parents of adolescents and identified a limited number of programs (eight programs in ten articles). Six programs addressed depressive disorders and/or related problems (21, 22, 32–34, 36, 37), and five addressed anxiety disorders and/or related problems (22, 32–35, 37). Schizophrenia/psychosis was addressed in two programs (23, 33, 34, 38), and only one program addressed all three mental health problems (33, 34). We did not perform a meta-analysis due to high ROB for observed in included studies, and high clinical and methodological heterogeneity across studies. Several studies found significant improvements in knowledge of mental health/illnesses and confidence and/or knowledge in helping children with mental health problems, while no studies found significant reduction in stigma toward people with mental health problems.

### Quality of Studies

#### Risk of Bias

ROB was “high” in the majority of included studies and “unclear” or “some concerns” in some others, for the following reasons. First, some of the descriptions of methods and results appeared to be unclear or lacking (21–23, 32–36, 38), and were not always clear enough to judge whether or not the outcomes were improved. More transparent reporting of the methods and results is needed. Second, measures such as likelihood-based methods, multiple imputation, and sensitivity analysis were not employed to reduce bias due to missing outcome data in the statistical analyses (21, 32). Third, no confounding variables were controlled for in the analyses in nonrandomized trials (23, 37, 38). Future studies could include covariates such as age, gender, and educational background (23, 39). In addition, intervention and control groups were from different areas in some studies (22, 37), which should be avoided. Finally, participants appeared to be aware of their assignment to intervention or control groups (21–23, 32–38). This also elevated ROB, according to the criteria (28, 29), but might be impossible to avoid in studies of education programs, unlike in tests of medications.

#### Other Issues

Participation rates were unknown in most studies (21–23, 33, 34, 36, 37). Most of these studies recruited some or all of their participants from online communities (e.g., via social media and website pages), and did not report the number of people who received information or were asked to participate in the study, which is needed to calculate the participation rate (21, 22, 33, 34, 36, 37). The following methods could help count the number of people who received the information: recruiting participants from parents at workplaces (32) or asking parents of students at schools to participate in the study. Next, some studies did not indicate whether the assessment questionnaires had been validated in regular people (21–23, 32, 35, 37). Lastly, sample sizes were small, for example, *n* < 100 in several studies (32, 35, 37). Larger studies will be needed in the future to draw more robust conclusions.

### The Effectiveness of MHL Programs

Although the evidence level for effectiveness of the MHL programs was low due to inconsistent quality, improvements in each outcome may be summarized as follows. Several programs might improve knowledge of mental health/illnesses (32–35, 37), and confidence and/or knowledge in helping children with mental health problems (32, 33). However, effect sizes in those studies were small to moderate (32–34); further studies are needed to confirm these effects. No programs appeared to reduce stigma toward people with mental health problems (22, 33, 34, 37, 38). Intention (22) / behavior (33, 34) of helping children with mental health problems were investigated in few studies. Future studies need to investigate these outcomes to clarify whether MHL programs have any actual impact on these parental behaviors.

No findings of reduction of stigma toward people with mental health problems in the included studies could be partly related to use of indirect measurement tools (38) or floor effects (22, 37). In one study (38), the extent to which an individual believes that most people would look down on or discriminate against people with schizophrenia was measured as stigma (38, 40); it may be difficult to change this kind of belief about others' stigmatizing attitudes through this kind of intervention. Assessments better matching to the purpose of interventions should be used. In two other studies (22, 37), personal stigma in the parents was low at baseline, with little room for the measured scores to improve.

When stratified by delivery mode, most face-to-face programs (33–35, 37) had a significant effect on knowledge of mental health/illnesses. However, effects were not clear for the online programs, due to unclear reports of the statistical results (36) and of the methods of statistical analyses (23). The effects of delivery mode on confidence were also not clear due to the limited number of studies (32–34, 36) or unclear reporting (36). Further comprehensive studies are needed to evaluate the effects of online programs, as well as face-to-face programs.

### Recommendation for Future Research

Educational settings may be the ideal place to implement MHL programs for parents. Implementing the programs at schools would enable sharing of the understanding of adolescent mental health, given that both schools and parents can play an important role in meeting the MHL needs of adolescents (19). Through this shared understanding, parents may more easily initiate a conversation with the school about any mental health concerns they have for their child; the reverse is also true. Schools may additionally provide informational resources for adolescents and their parents, as well as arrange access to professional care through school counselors or health centers, lowering barriers to help-seeking.

In future programs, a focus on family-based approaches may be beneficial. Sharing of attitudes toward and knowledge of mental health between parents and children could reduce barriers to treatment, and development of programs that they can participate in together should be considered. In addition to improvements in studies of parental MHL programs, concerted efforts need to be made by researchers, as well as policy makers, to raise awareness of the importance of MHL for both parents and their children. For example, researchers could reach out to educational institutions as well as educational ministries/boards to encourage collaborative development of MHL programs. Considering the increasing prevalence of mental illnesses, and the high burden on youth and their caregivers (parents), health care systems, as well as society at the whole, these are issues must be given urgent attention.

### Limitations

First, studies from sources other than scientific databases such as non-profit organizations and governments may have been overlooked. Second, relevant studies not written in English were also not examined. Third, although we tried to obtain information that was missing or unclear in the included studies, not all authors were available. Finally, publication bias was not assessed, given inadequate amounts of comparable data due to the variety of measurement tools used in the included studies.

## Conclusions

The quality of the literature assessing effectiveness of previously developed MHL programs in parents of adolescents was inconsistent. Therefore, it remains unclear whether the programs overall were truly effective in improving parental MHL. However, significant positive effects were shown in several studies. It appears useful and worthwhile to develop educational programs to support parental MHL, although higher quality studies with clearer and more transparent reporting are needed. For example, in-depth description of details such as participation rates, methods, statistical analyses and outcomes are necessary. The effects on actual helping behavior in parents need to be measured in more studies.

## Data Availability Statement

The original contributions presented in the study are included in the article/supplementary material, further inquiries can be directed to the corresponding author/s.

## Author Contributions

SK and TS designed the study, and wrote the review protocol with SY, JF, and FT. SK and SY carried out the study selection and the assessment of risk of bias. SK also conducted the data extraction, and SY confirmed the extracted data. TS was the third reviewer to help the study selection, the assessment of risk of bias and the data extraction. SK drafted the study, and TS, FT, JF, and SY revised the draft. All authors approved the final manuscript.

## Funding

This study was supported by grants from the Japan Society for the Promotion of Science (JSPS KAKENHI Grant Numbers 18H01009, 21H00857, and JP21J21319).

## Conflict of Interest

The authors declare that the research was conducted in the absence of any commercial or financial relationships that could be construed as a potential conflict of interest.

## Publisher's Note

All claims expressed in this article are solely those of the authors and do not necessarily represent those of their affiliated organizations, or those of the publisher, the editors and the reviewers. Any product that may be evaluated in this article, or claim that may be made by its manufacturer, is not guaranteed or endorsed by the publisher.

## Abbreviations

CBA: controlled before-and-after study
Con: control group
Int: intervention group
ROB: risk of bias
w: week
y: year.
